# Serum Uric Acid as a Diagnostic Biomarker for Rheumatoid Arthritis–Associated Interstitial Lung Disease

**DOI:** 10.1007/s10753-022-01661-w

**Published:** 2022-03-22

**Authors:** Zitao Wang, Wen Wang, Ting Xiang, Bangdong Gong, Jianmin Xie

**Affiliations:** 1 Department of Rheumatology and Immunology, The Second Affiliated Hospital of Nanjing Medical University, Nanjing, People’s Republic of China; 2 Department of Rheumatology and Immunology, Shanghai Tongji Hospital, Shanghai, China

**Keywords:** Rheumatoid arthritis, Uric acid, Interstitial lung disease, Epithelial-mesenchymal transition

## Abstract

Previous studies have suggested a correlation between uric acid (UA) and lung lesion in some diseases. However, it remains unknown whether UA contributes to the lung injury in rheumatoid arthritis (RA). Our study aimed to investigate the clinical value of the UA level in the severity of rheumatoid arthritis–associated interstitial lung disease (RA-ILD). We measured UA in serum and bronchoalveolar lavage fluid (BALF), and UA levels of subjects were compared. As for the role of UA on ILD, we incubated A549 cells with UA and the expression of EMT markers was measured by immunofluorescence staining. The concentrations and messenger RNA expression of IL-1, IL-6, and transforming growth factor-β (TGF-β) were measured by ELISA and RT-PCR, respectively. We observed that serum UA levels in RA were significantly higher than those in controls. And, higher UA was measured in both serum and BALF of patients with RA-ILD, particularly those with interstitial pneumonia (UIP) pattern. Additionally, the correlation of the serum and BALF UA levels with serum KL-6, a biomarker of ILDs, in RA was significant (*r* = 0.44, *p* < 0.01; *r* = 0.43, *p* < 0.01). And, the negative correlations of UA, in both serum and BALF, with forced vital capacity (*r* =  −0.61, *p* < 0.01; *r* =  −0.34, *p* < 0.01) and diffusing capacity for carbon monoxide (*r* =  −0.43, *p* < 0.01; *r* =  −0.30, *p* < 0.01) were measured in patients. In the ROC curve analysis, the AUC value of UA for RA-ILD was 0.744 (95% CI: 0.69–0.80; *p* < 0.01), and the AUC of serum UA for predicting UIP pattern of patients with RA-ILD was 0.845 (95% CI: 0.78–0.91; *p* < 0.01), which showed the significance of the UA in clinical settings. Also, the in vitro experiment showed that UA induced epithelial-to-mesenchymal transition (EMT) and production of IL-1, IL-6, and TGF-β in A549 cells. Therefore, the elevated UA levels may be a diagnostic marker in RA-ILD, particularly RA-UIP.

## INTRODUCTION

Uric acid (UA) is a final catabolite of purine found in organisms. In recent years, interest in uric acid as a regulator of inflammation and immune responses has grown [1, 2]. Previous publications suggested that UA has the ability to stimulate the expression of C-reactive protein (CRP), fibrinogen, and complement C3 in a dose-dependent manner [3]. Growing evidence indicates that serum uric acid is closely associated with circulating levels of interleukin-6 (IL-6), CRP, and tumor necrosis factor-α (TNF-α), which implies that UA may play a critical role in systemic inflammation and inflammatory-related diseases, such as rheumatoid arthritis (RA) [4]. Moreover, a series of studies have reported that uric acid crystals are not just innocent bystanders but may be active players in the proliferation and differentiation of T cells that participate in the pathology of inflammatory disorders and autoinflammatory diseases. The experimental data demonstrated that UA coordinates with inflammasome-dependent cytokines contribute to the differentiation of Th17 cells both *in vitro* and *in vivo* [5]. The correlation between Treg cell numbers and UA in systemic lupus erythematosus (SLE) has also been reported [6]. In addition, UA has been confirmed to be a considerable initiator and amplifier of Th2 cell function during immunity and allergic inflammation and to be an inflammatory mediator in allergic asthma [7]; additionally, it has been proposed that the local accumulation of UA, as an endogenous danger signal, can activate the Nod-like receptor protein 3 (NALP3) inflammasome, promoting the production of IL-1β, thereby contributing to lung inflammation and fibrosis [8]. Of note, increasing evidence has shown that a high level of UA, which is associated with both the presence of pulmonary hypertension (PH) and higher mortality, has prognostic value in ILD [9]. Recent studies verified that uric acid crystals formed at the lung injury site might represent a key danger signal for activating the inflammasome to release inflammatory cytokines, thereby causing inflammatory lung pathologies [8]. Moreover, increased serum UA concentrations in patients with lung disease of different etiologies correlate with the prognosis and severity of the disease [10–13].

RA is a chronic systemic inflammatory disease characterized by progressive and erosive destruction by arthritis accompanied by damage to multiple organs [14]. Interstitial lung disease (ILD), the most common extra-articular complication of RA, is associated with significant mortality and is a key factor affecting the prognosis of patients with RA [15, 16]. Since RA-associated ILD (RA-ILD) has a high mortality rate, clarifying its pathogenesis and treatment strategies is necessary. Recently, some documents confirmed a novel role for the danger signal uric acid in driving proinflammatory Th17 differentiation, and significant relationships of systemic sclerosis (SSc) with pulmonary fibrosis and elevated UA levels have been reported [17]. Although the potential function of UA in lung disease has increasingly appreciated, there is little information available on the clinical significance of UA in patients with RA and ILD.

Therefore, we hypothesize that UA might be engaged in the process of RA and is associated with the development of RA-ILD, and an assessment of UA may have clinical implications for identifying ILD with usual interstitial pneumonia (UIP) patterns in patients with RA.

## MATERIALS AND METHODS

### Study Population

In this study, 207 consecutive patients (> 18 years old) with a primary clinical diagnosis of RA who fulfilled the American College of Rheumatology/European League Against Rheumatism 2010 diagnostic criteria and 59 patients who have accepted treated in other hospitals were recruited from the Second Affiliated Hospital of Nanjing Medical University between January 2019 and December 2020. We excluded patients suffering from other autoimmune diseases, such as SLE, systemic scleroderma, and dermatomyositis (DM), acute infectious disease, gout, and severe hepatic and renal dysfunction, or those with oncological diseases and heart failure as well as taking uric acid–lowering drugs and other drugs affecting uric acid levels. General condition, past medical history, smoking status, and duration of RA were obtained from medical clinical records. The ILD of all patients collected in this study was defined according to high-resolution computed tomography (HRCT) evidence. Meanwhile, partial patients were evaluated with pulmonary function tests (PFTs) and bronchoalveolar lavage (BAL). Additionally, 138 individuals matched by age and sex were also included as healthy controls (HCs) from healthy staff members of the hospital. The clinical characteristics and demographic profiles of the subjects are presented in Table 1.

**Table 1:** Demographic, Clinical, and Laboratory Characteristics of Subjects

Characteristics	HC (*n* = 138)	All (*n* = 266)			RA-ILD (*n* = 162)		
		No-RA-ILD (*n* = 104)	RA-ILD (*n* = 162)	*p* value	RA-NSIP (*n* = 86)	RA-UIP (*n* = 76)	*p* value
General demographics							
Sex (male/female) (%female)	26/112 (81.2)	20/84 (80.8)	28/134 (82.7)	0.752	15/71 (82.6)	13/63 (82.9)	0.781
Age (mean ± SD, years)	66.04 ± 13.62	63.85 ± 13.20	54.66 ± 3.94	0.823	52.44 ± 2.73	57.18 ± 3.56	0.462
Duration of the disease (months)	–	58 (2–256)	62 (3–360)	0.607	63 (6–360)	62 (1–360)	0.581
Smoking history, *n* (%)	34 (24.6)	11 (10.5)	51 (19.2)	0.048	8 (9.3)	43 (56.5)	0.036
Laboratory values							
RF (IU/mL)	–	103.04 ± 162.14	235.31 ± 182.76	0.012	156.50 ± 81.20	324.50 ± 220.80	0.009
Anti-CCP (RU/mL)	–	326.38 ± 361.04	467.84 ± 375.95	0.015	367.30 ± 235.60	581.60 ± 462.70	0.004
ESR (mm/h)	–	28.59 ± 24.28	50.31 ± 8.51	0.009	48.27 ± 6.74	52.61 ± 9.64	0.367
CRP (mg/L)	–	14.63 ± 17.68	63.00 ± 20.59	0.001	46.75 ± 4.85	81.39 ± 15.49	0.036
UA (µmol/L)	–	252.38 ± 6.15	291.81 ± 102.42	0.002	262.86 ± 103.45	475.58 ± 249.28	0.001
KL-6 (U/mL)	–	395.97 ± 216.39	910.06 ± 955.24	0.001	778.86 ± 689.51	1058.53 ± 1168.55	0.003
Current treatments							
NSAIDs	–	25 (24.0)	29 (17.9)	0.247	18 (20.9)	11 (14.5)	0.471
Glucocorticoids	–	20 (19.2)	39 (24.1)	0.549	23 (26.7)	16 (21.1)	0.168
MTX	–	21 (21.1)	29 (17.9)	0.624	17 (19.8)	12 (15.8)	0.652
Bio-DMARDs	–	22 (21.2)	24 (14.8)	0.218	15 (17.4)	9 (11.8)	0.536
Clinical features							
RA duration (mean ± SD) (years)	–	6.56 ± 4.28	7.45 ± 4.61	0.695	5.46 ± 2.62	9.71 ± 5.28	0.153
DAS28 score	–	5.52 ± 1.87	6.62 ± 2.00	0.395	6.45 ± 1.25	6.82 ± 2.58	0.421
Cough	–	12 (11.5)	55 (34.0)	< 0.01	31 (36.0)	24 (31.6)	0.549
Sputum	–	9 (8.7)	27 (16.7)	0.062	17 (19.8)	10 (13.2)	0.260
Dyspnea	–	0 (0)	34 (21.0)	< 0.01	15 (17.4)	19 (25.0)	0.238
Chest congestion	–	0 (0)	28 (17.3)	< 0.01	11 (12.8)	17 (22.4)	0.108
Dry rale	–	0 (0)	38 (23.5)	< 0.01	17 (19.8)	21 (27.6)	0.238
Bluish skin tinge	–	0 (0)	3 (1.9)	0.163	2 (2.3)	1 (1.3)	0.634
Clubbing	–	0 (0)	3 (1.9)	0.163	3 (3.5)	0 (0)	0.12

*HC* healthy control, *RF* rheumatoid factor, *CCP* cyclic peptide containing citrulline, *ESR* erythrocyte sedimentation rate, *CRP* C-reactive protein, *UA* uric acid, *KL-6* Krebs von den Lungen-6, *NSAIDs* nonsteroidal anti-inflammatory drugs, *DMARDs* disease-modifying anti-rheumatic drugs, *DAS28* 28-Joint Disease Activity Score

All subjects who participated in this study voluntarily provided written informed consent. The study protocol was approved by the medical ethics committee of the hospital.

### Clinical and Laboratory Assessments

We registered the patients’ general condition, including age, sex, duration of RA, and comorbidities. Laboratory indexes such as CRP, erythrocyte sedimentation rate (ESR), presence and plasma level of rheumatoid factor (RF), and anti-cyclic peptide containing citrulline (anti-CCP) were collected. Serum samples of patients were analyzed for UA level and the concentration of Krebs von den Lungen-6 (KL-6). All basic demographic and clinical characteristics of the participants are shown in Table 1.

### Bronchoalveolar Lavage

Based on current evidence, BAL cellular analysis might be a supplementary means for the diagnostic evaluation of ILD. In this study, BAL was performed in 97 patients with RA (58 RA-ILD patients and 39 non-RA-ILD patients, respectively). Bronchoalveolar lavage fluid (BALF) was collected, and then UA concentrations were measured as described previously [18].

### Measurement of KL-6

The concentrations of KL-6 in serum were measured by an enzyme-linked immunosorbent assay (ELISA) kit according to previously described procedures [19].

### Radiographic Classification

According to the American Thoracic Society/European Respiratory Society (ATS/ERS) classification of idiopathic interstitial pneumonias (IIPs) [20, 21], the HRCT patterns of RA-ILD were categorized into two major subgroups, namely UIP and nonspecific interstitial pneumonia (NSIP). Separately, the UIP pattern was characterized by traction bronchiectasis/bronchiectasis, reticular opacity, and honeycombing, while the HRCT characteristic of NSIP was extensive ground glass opacity [22]. HRCT scans of each patient were independently reviewed by two trained thoracic radiologists who were blinded to all clinical information such as demographics and general state. Disagreements over the diagnosis were resolved by consensus. According to the classification method proposed by Ichikado *et al.* [23], the HRCT findings were scored.

### Pulmonary Function Tests

Patients with RA-ILD who underwent PFTs fulfilled the ATS/ERS guidelines [24–26], including forced vital capacity (FVC) and diffusing capacity for carbon monoxide (DLCO), which were conducted for all patients. DLCO values of 80% of predicted normal values and FVC values of 80% of predicted normal values were considered abnormal and also recorded. All the data are presented as percentages of the normal predicted values. PFTs were not performed except in those patients with clinical features of ILD (*n* = 72).

### Cell Culture, Treatment, and Analysis

A549 cell line was used to resemble type II alveolar epithelial cells [27]. A549 cells were cultured in vitro. Before UA treatment, A549 cells were maintained in serum-free media for 12 h prior to stimulation and then changed to complete media with increasing UA concentrations of 0 µmol/L, 200 µmol/L, and 400 µmol/L for 24 h. Supernatants were then collected, and IL-1, IL-6, and transforming growth factor-β (TGF-β) concentrations were determined by ELISA.

### Real-Time Reverse Transcription Semiquantitative Polymerase Chain Reaction

Total RNA was extracted from A549 cells by TRIzol® RNA isolation (Gibco, Thermo Fisher Scientific, Inc.) and purified with DNase I (Invitrogen, Thermo Fisher Scientific, Inc.) according to the manufacturer’s protocol. The primer sequences are available upon request. RNAs were reverse-transcribed into cDNA using SuperScript™ II (Invitrogen Life Technology). Real-time quantitative PCR was performed by fluorescent dye SYBR Green methodology using SYBR Green PCR Master Mix (Applied Biosystems) and the ABI Prism 7000 apparatus (Perkin-Elmer, Foster City, CA, USA). Gene expression was normalized to the corresponding β-actin level and is presented as the fold change relative to that of the control.

### Immunofluorescence Staining

A549 cells were seeded in 6‑well plates. After the indicated treatment, the cells were fixed and incubated with primary antibodies against α-SMA (Santa Cruz Biotechnology, Inc.) and E‑cadherin (Santa Cruz Biotechnology, Inc.) overnight at 4 °C, after which the cells were washed three times with PBS. The cells were stained as described in previous studies [28].

### Statistical Analysis

All statistical analyses were carried out using GraphPad Prism version 8.0.2. Differences between groups were analyzed by Student’s *t* test. Comparisons of categorical variables were conducted using Pearson chi-square tests. For nonparametric data, the results were expressed as median (range) values, and the differences between groups were analyzed by the Mann–Whitney *U* test. Spearman correlation analysis was performed to analyze the association of UA with clinical and laboratory indexes in patients with RA. Univariate logistic regression analysis was performed to determine the factors associated with the presence of ILD. Multivariate logistic regression analysis was performed by including the confounding factors that were found to be significantly associated with the univariate analyses. The receiver operating characteristic (ROC) curve and area under the curve (AUC) were used to assess the validity and value of UA for RA-ILD. Any difference with a *p* value < 0.05 was regarded as statistically significant.

## RESULTS

### Relationship of Serum UA Level with Laboratory Indexes of Patients with RA

**Fig. 1:**
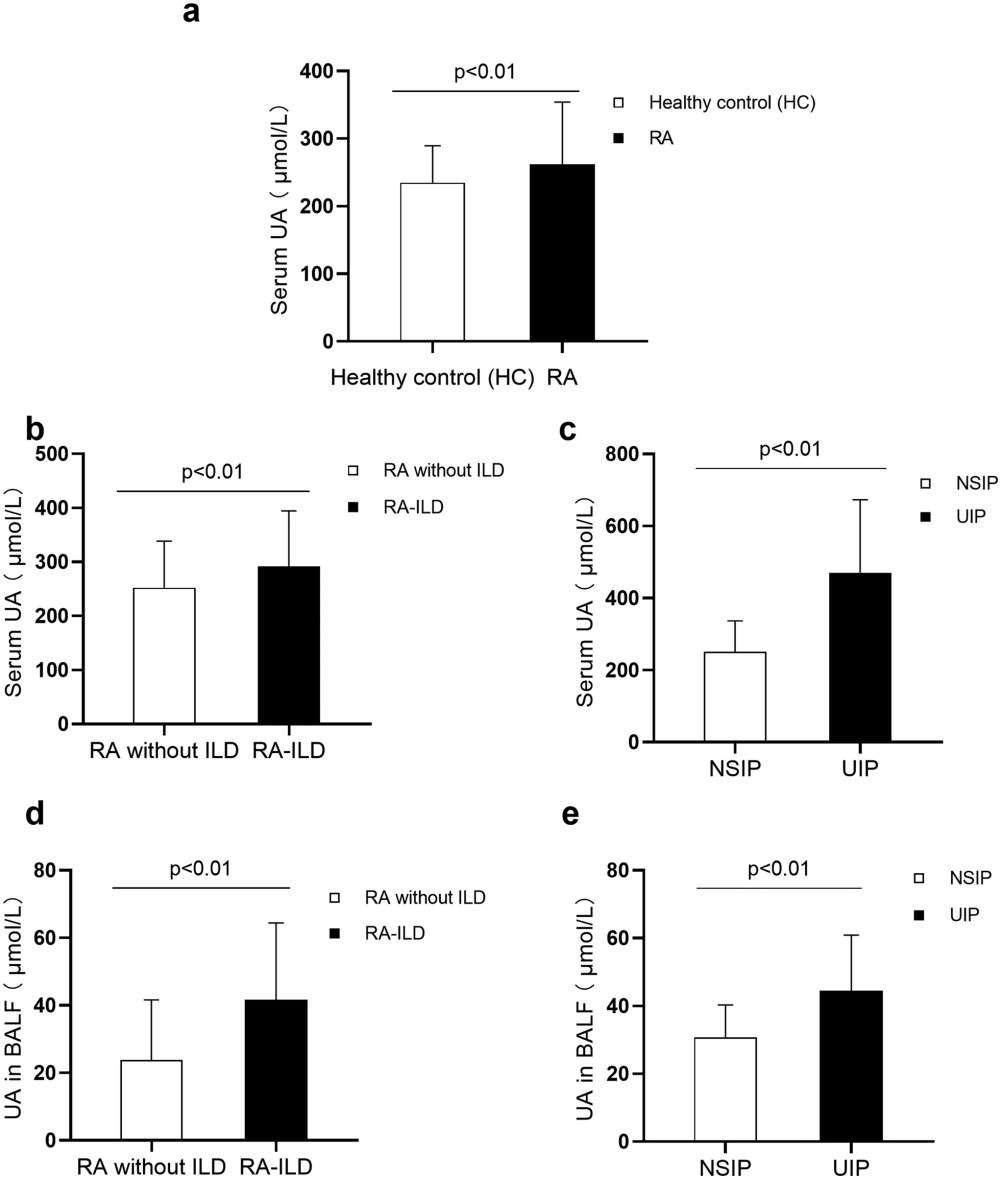
The level of UA was measured in patients with RA-ILD. The level of UA in the serum of patients with RA (*n* = 266) and healthy control (HC) (*n* = 138). Statistical difference was detected between patients with RA and HC (*p* < 0.01) **a**. Comparison of serum UA levels between non-ILD and RA-ILD, as well as NSIP and UIP. The higher serum UA levels were observed in patients with RA-ILD (*n* = 162) relative to RA patients without ILD (*n* = 104) (*p* < 0.01) **b**. Statistical difference of serum UA was also detected between UIP (*n* = 76) and NSIP (*n* = 86) patterns of RA-ILD (*p* < 0.01) **c**. The UA level in BALF of patients with RA (*n* = 97) was also measured. Compared with RA patients without ILD (*n* = 39), the level of UA in patients with RA-ILD (*n* = 58) was significantly higher (*p* < 0.01) **d**. And, a marked increase of the level of UA was observed in BALF in patients with RA-UIP (*n* = 37) relative to RA patients with NSIP pattern (*n* = 21) (*p* < 0.01) **e**.

**Fig. 2:**
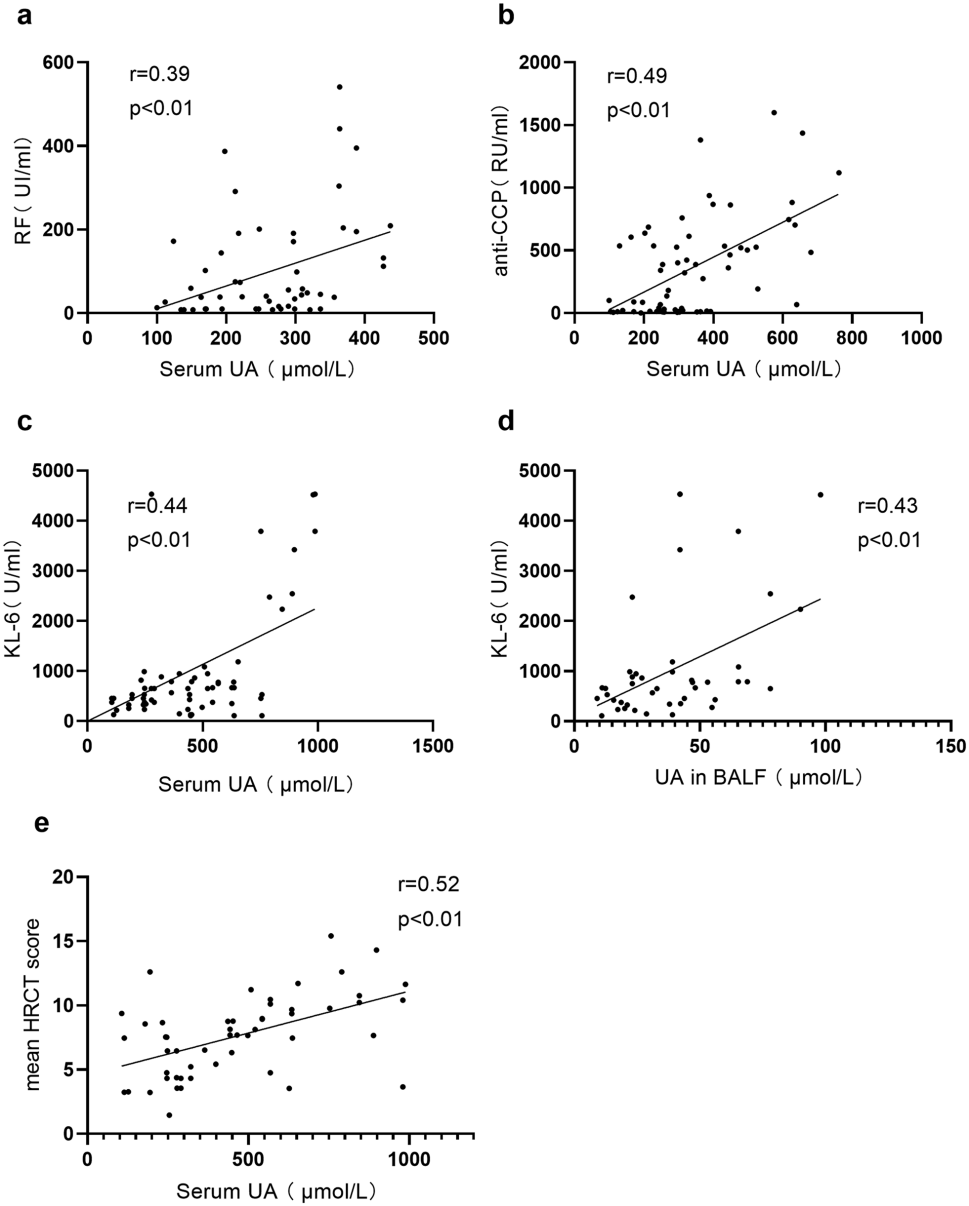
Correlation of UA with clinical and laboratory indexes in patients with RA. RF and anti-CCP were positively correlated with the serum UA in patients with RA (*n* = 63) **a**, **b**. A positive correlation between serum UA and KL-6 was detected by the Spearman test (*n* = 62) **c**. UA in BALF was positively associated with KL-6 in RA **d**. Serum UA also showed a positive correlation with the mean HRCT score **e**.

The demographic characteristics, the UA level in serum, and other laboratory values of patients with RA are presented in Table 1. We did not find a significant association between current treatments and subtypes of ILD in patients with RA. But, whether this is related to the insufficient sample size of patients receiving ongoing treatment included in our current study needs to be further verified in future studies. To investigate the relationship between the serum levels of UA and RA, we compared the demographics and serum UA level of the RA and control groups. The serum UA level in the patients with RA was significantly higher than that in the healthy controls (262.25 ± 91.94 µmol/L *vs.* 234.69 ± 54.78 µmol/L, *p* < 0.01) (Fig. 1a). As shown in the Spearman correlation analysis, serum UA levels were positively correlated with RF and anti-CCP in patients with RA (*r* = 0.39, *p* < 0.01; *r* = 0.49, *p* < 0.01) (Fig. 2a, b). Next, we evaluated the strength of the association between serum UA and CRP and ESR. The results showed that the serum UA level was apparently correlated with CRP and ESR (*r* = 0.26, *p* < 0.01; *r* = 0.27, *p* < 0.01).

### The UA Level Was Elevated, Especially in Patients with RA and ILD, and Correlated with the KL-6 Level

Based on the above data of the RA and healthy groups, as well as the Spearman correlation analysis results, we concluded that UA may be an important biomarker of RA. Furthermore, we explored whether the UA level was associated with RA-ILD in patients with RA. Patients were divided into the RA-ILD group (*n* = 162) and the non-RA-ILD group (*n* = 104). The level of serum UA was significantly higher in patients with RA-ILD than in those without ILD (291.81 ± 102.42 µmol/L *vs.* 252.38 ± 6.15 µmol/L, *p* < 0.01) (Fig. 1b). In addition, we also measured the concentration of UA in BALF of patients with partial RA. The results show that more UA was detected in the BALF of the RA-ILD group than in the BALF of the non-ILD group (41.7 ± 22.7 µmol/L *vs.* 23.8 ± 17.8 µmol/L, *p* < 0.01) (Fig. 1d). To further verify that UA levels were associated with lung injury markers in patients with RA-ILD, we subsequently measured the concentration of KL-6, a biomarker of ILDs [29], in the serum of patients with RA and examined the relationship between the levels of UA and KL-6 by correlation analysis. The results showed that serum UA levels positively correlated with KL-6 in patients with RA (*r* = 0.44, *p* < 0.01) (Fig. 2c). Similarly, the UA level in BALF also had a powerful correlation with serum KL-6 in patients with RA (*r* = 0.43, *p* < 0.01) (Fig. 2d).

### High Serum and BALF UA Levels Were Correlated with the UIP Pattern and the Progression of RA-ILD

**Fig. 3:**
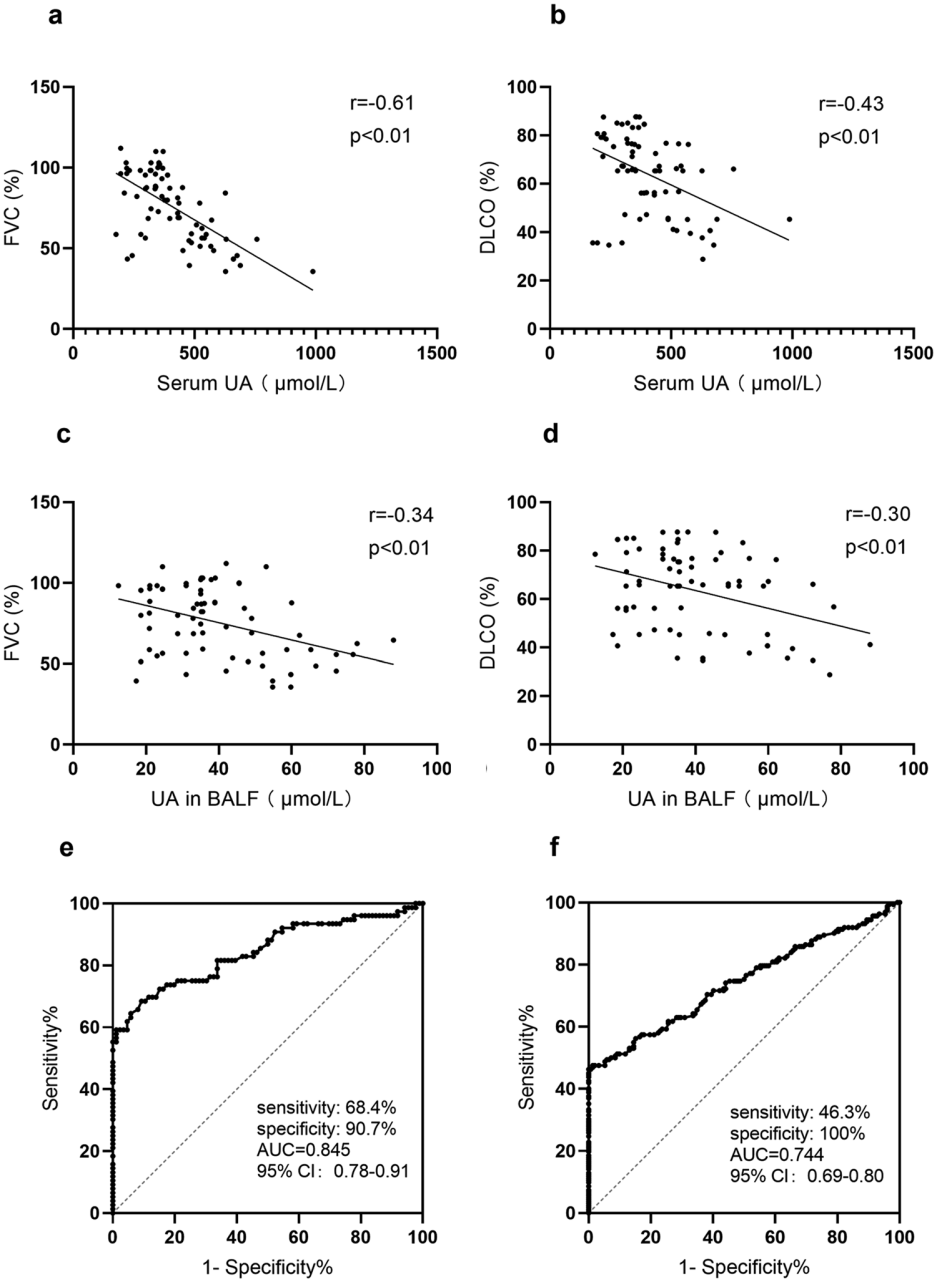
Correlations between the UA level in serum and BALF and lung function (*n* = 72). Serum UA levels were inversely correlated with FVC **a** and DLCO **b** in patients with RA-ILD. And, UA levels in BALF negatively correlated with pulmonary function indexes **c**, **d**. Receiver operating characteristic (ROC) curve showing the predictive capacity of UA in the presence of UIP pattern in RA. The area under the ROC curve (AUC) was 0.76 (95% CI = 0.66–0.87; sensitivity = 60%; specificity = 92%; *p* < 0.01) **e**. ROC curve analysis of serum UA in the RA-ILD group with an AUC of 0.744 (*p* < 0.01). Using a cutoff level of 320 µmol/L **f**.

The above data showed that the UA level was higher in RA patients with ILD than in patients without ILD. Clinically, RA-ILD manifests many well-recognized phenotypes, in which the UIP pattern is the most common form of RA-ILD with a worse prognosis [16]. Thus, the distinction of UIP in patients with RA-ILD is of important clinical value for guiding the treatment of patients with RA-ILD. In view of the aforementioned findings of UA levels in RA-ILD, we hypothesized that UA levels may be associated with the UIP pattern and the progression of RA-ILD. Based on the HRCT scans, 76 subjects with UIP patterns and 86 subjects with NSIP patterns were identified among the patients with RA-ILD. Compared with the NSIP group, the level of serum UA in RA-ILD patients with the UIP pattern was markedly higher (475.58 ± 249.28 µmol/L *vs.* 262.86 ± 103.45 µmol/L, *p* < 0.01) (Fig. 1c). We performed BAL in 58 patients with RA-ILD. Interestingly, the UA level in BALF was significantly higher in RA-UIP patients than in RA-ILD patients with the NSIP pattern (44.5 ± 16.4 µmol/L *vs.* 30.7 ± 9.5 µmol/L, *p* < 0.01) (Fig. 1e). To investigate whether the UA level was correlated with a decline in lung function in patients with RA-UIP, we evaluated the lung function of RA-ILD patients with the UIP and NSIP patterns. The results showed that the UA levels in serum and BALF were negatively correlated with FVC (*r* =  −0.61, *p* < 0.01; *r* =  −0.34, *p* < 0.01) (Fig. 3a, c) and DLCO% predicted (*r* =  −0.43, *p* < 0.01; *r* =  −0.30, *p* < 0.01) (Fig. 3b, d). These data suggest that UA may be a significant contributor to the pathogenesis of RA-ILD.

### Serum UA Levels Can Be a Disease Marker for the Detection of RA-ILD

To evaluate the diagnostic value of the serum UA level in clinical settings, we established a ROC curve to quantify the predictive ability of serum UA level for RA-ILD (AUC = 0.744, *p* < 0.001) (Fig. 3f). In parallel, we investigated the diagnostic performance of serum UA for identifying RA patients with UIP. The AUC was 0.845 (95% CI: 0.78–0.91; *p* < 0.001). And, the cutoff value was 384.5 µmol/L, with a sensitivity and specificity of 68.4% and 90.7%, respectively (Fig. 3e). Thus, these results suggest that serum UA may be a positive biomarker for distinguishing the ILD in patients with RA, especially the UIP pattern, which means that it can be a significant biomarker candidate for appraising the disease progression and severity of ILD in patients with RA.

### UA Induces Epithelial-to-Mesenchymal Transition in A549 Cells

**Fig. 4:**
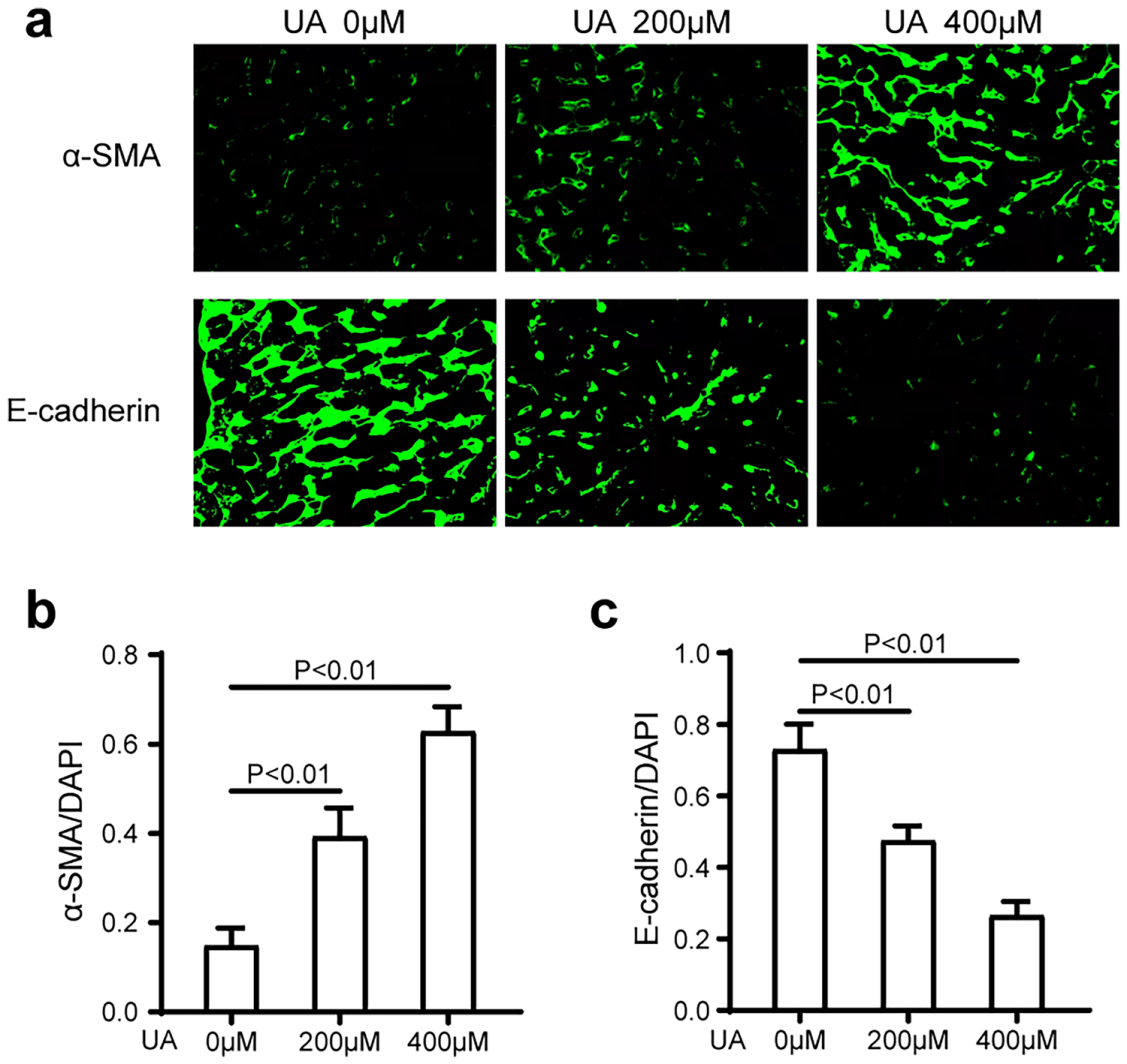
UA induces EMT in primary human lung alveolar type II (A549) cells. UA augmented the expression of the mesenchymal cell marker α-SMA in a dose-dependent manner (*p* < 0.01) **a**, **b**. In contrast, UA decreased the expression of the epithelial cell marker E-cadherin in a dose-dependent manner (*p* < 0.01) **a**, **c**. The fluorescent images were obtained by a confocal laser scanning microscope.

Emerging evidence has demonstrated that epithelial-to-mesenchymal transition (EMT) phenomena are potential contributors to ILD, that alveolar epithelial cells from interstitial lung diseases undergo epithelial-to-mesenchymal transitions after lung injury, and that these alveolar epithelial cells undergo EMT, which includes loss of their epithelial biomarkers and acquisition of mesenchymal (fibroblast-like) cell biomarkers [30]. To assess whether UA could induce EMT in A549 cells, we performed immunofluorescence staining of EMT markers, including E-cadherin and α-SMA, after A549 cells were incubated with UA (0 µM, 200 µM, and 400 µM) for 24 h. We found that UA decreased the expression of the epithelial cell marker E-cadherin in a dose-dependent manner (Fig. 4a, b). In contrast, UA augmented the expression of the mesenchymal cell marker α-SMA in a dose-dependent manner (Fig. 4c). These findings revealed that high UA was shown to induce EMT in A549 cells.

### The Production of Cytokines Was Induced by High UA in A549 Cells

**Fig. 5:**
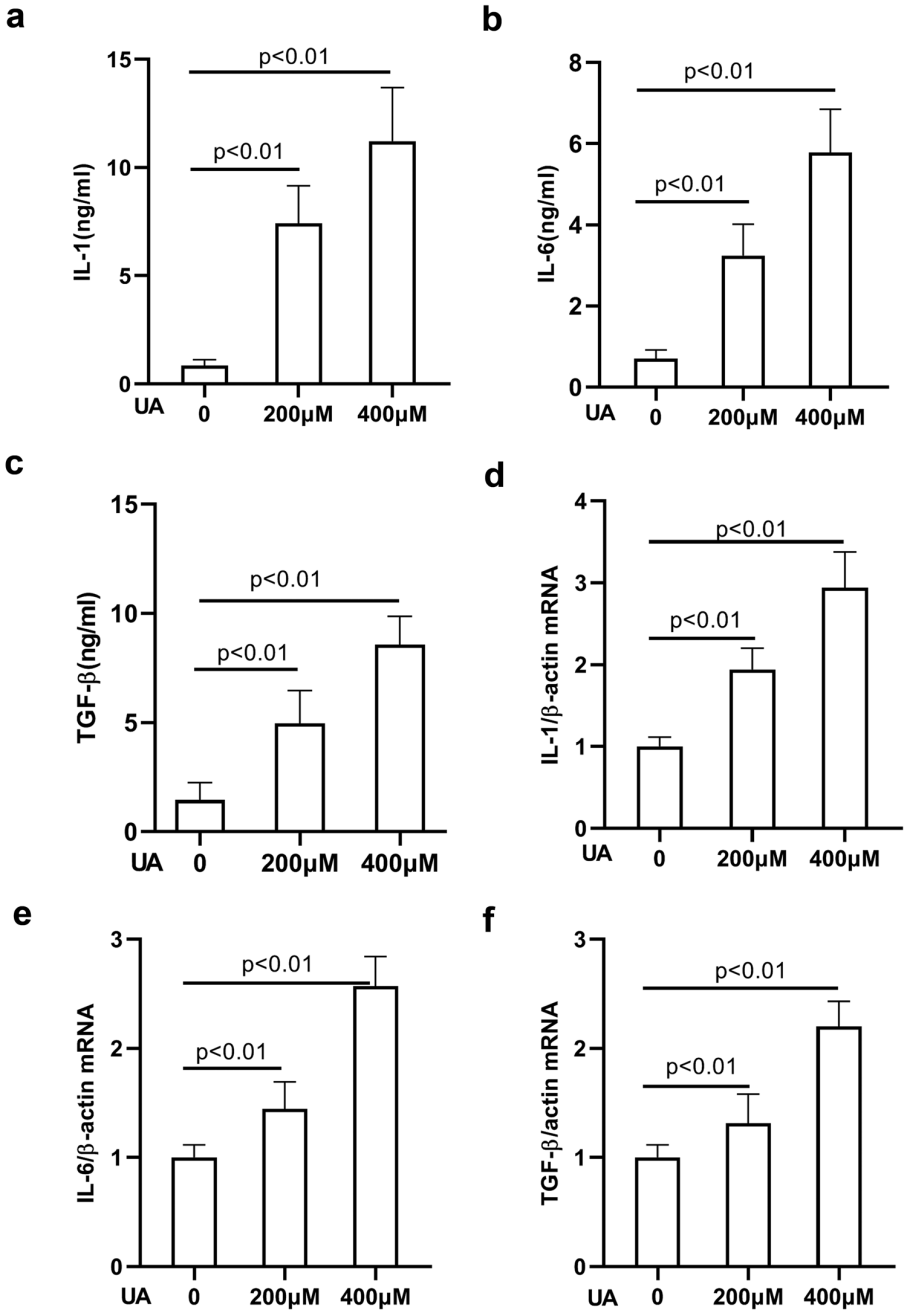
UA induces the production of cytokines in A549 cells. Supernatants of A549 cells stimulated with UA were harvested for ELISA analysis. After a 24-h treatment, the production of IL-1, IL-6, and transforming growth factor-β (TGF-β) by A549 alveolar epithelial cells was markedly increased by UA in a dose-dependent manner compared with that of the control group (*p* < 0.01) **a**–**c**. A549 cells treated with increasing concentrations of UA were also harvested for quantitative RT-PCR analysis. mRNA expression of IL-1, IL-6, and TGF-β was upregulated by UA in a dose-dependent manner compared with that of the control group (*p* < 0.01) **d**–**f**.

It has been demonstrated that these proinflammatory cytokines exert direct proinflammatory effects on alveolar and bronchial epithelial cells, which are well-known lung pathology promoters [31, 32]. Next, we explored whether UA is capable of stimulating the release of cytokines from A549 cells. A549 cells were treated with increasing concentrations of UA (0 µM, 200 µM, and 400 µM), and supernatants of A549 cells stimulated with UA were harvested for ELISA analysis. As shown in Fig. 5a–c, the production of IL-1, IL-6, and TGF-β by A549 alveolar epithelial cells was markedly increased by UA in a dose-dependent manner compared with that of the control group, both at 24 h after treatment. A549 cells treated with increasing concentrations of UA were also harvested for quantitative RT-PCR analysis. As shown in Fig. 5d–f, the mRNA expression of IL-1, IL-6, and TGF-β was upregulated by UA in a dose-dependent manner compared with that of the control group, both at 24 h after treatment. These results revealed that high UA could induce the expression of cytokines in A549 cells.

## DISCUSSION

This is the first report on the strong association between serum UA levels and the severity and progression of UIP in RA patients with ILD relative to RA patients without ILD. Furthermore, serum UA levels were significantly increased in the UIP pattern of patients with RA-ILD. Intriguingly, the increased serum UA level in patients with RA-ILD was positively correlated with the HRCT-UIP score (Fig. 2e) and RF. ROC curves also showed that the serum UA level may be an accurate biomarker for the presence of interstitial lung disease and the identification of UIP pattern in patients with RA. These data should suggest that the circulating UA concentration may be a potential biomarker for identifying ILD, especially UIP pattern, and evaluating the progression and severity of ILD in patients with RA.

UA is an antioxidant that is remarkable in the airway mucous membrane, and its actual role, especially in the regulation of lung function, has attracted attention in various diseases [18, 33]. The present study showed that the level of UA was significantly associated with RA and the presence of ILD in RA, and a higher UA level was measured in the serum of individuals with RA. Significant correlations between serum UA levels and other related laboratory parameters in RA, including RF and anti-CCP, were observed. We also found that smoking history showed significant differences in the RA groups with and without ILD, which was consistent with previous reports [34, 35]. Additionally, compared with RA patients without ILD, the levels of serum and BALF UA were robustly higher in patients with RA-ILD, suggesting that UA may have specific correlations with ILD in patients with RA. Furthermore, the serum UA level was elevated in the UIP pattern of patients with RA-ILD compared to the NSIP pattern of patients with RA-ILD, which indicated the apparent correlation between UA and the more severe UIP pattern of RA-ILD. Thus, we believe that UA plays an important role in the pathogenesis of RA-ILD.

Regarding the underlying work, we have confirmed that serum UA levels have an obvious association with RA-ILD, and circulating UA levels have a strong correlation with ILD structural damage (HRCT score) and a significant correlation with pulmonary function impairment (FVC, DLCO). Most notably, the circulating UA level has a remarkable association with the clinically severest RA-ILD disease phenotype, *i.e.*, the UIP pattern. The overall function of UA in terms of its association with ILD is apparently correlated with the currently available marker KL-6. The good predictability of the serum UA level implied a potential role as a new biomarker for RA-ILD.

Some experimental and clinical reports have confirmed that UA might be closely related to inflammatory responses, and serum UA levels are positively associated with TNF-α, IL-6, and CRP [18, 36]. Interestingly, the results of an in vitro experimental study revealed that monosodium urate crystals can activate human fibroblast-like synoviocytes (FLSs) from RA patients with a remarkable increase in the release of the inflammatory cytokine IL-6, which provided insight into the role of UA in provoking inflammation in RA [37]. In our study, the level of serum UA showed significant positive correlations with several inflammatory risk factors for RA, including CRP and ESR, suggesting that serum UA levels may be used as an effective indicator for the inflammatory activity of RA.

Interstitial lung diseases are characterized by destruction of lung architecture due to excessive deposition of extracellular matrix proteins by myofibroblast activation, leading to aggravated dyspnea and loss of lung function [38, 39]. Numerous studies have shown that EMT is involved in the development and progression of many lung diseases [40–42]. Further, EMT plays a critical role in the accumulation of myofibroblasts and the resultant deposition of extracellular matrix proteins, which is a crucial step in the progression of ILD [43]. Both EMT and aberrant phenotypic changes in alveolar epithelial cells are prominent for the progression of ILD [38, 44]. To further verify that UA is involved in the pathogenesis of ILD, we investigated whether UA contributed to the EMT of alveolar epithelial cells. Our data revealed that UA could significantly induce EMT in alveolar epithelial cells (A549 cells), including elevation of mesenchymal markers and decline of epithelial cell markers. In a recent study, soluble UA was found to contribute to lung interstitial fibrosis closely related to the NLRP3 inflammatory pathway [8]. In contrast, the consensus among the previous works showed that some proinflammatory cytokines, such as IL-1, IL-6, and TGF-β, secreted by alveolar epithelial cells are closely related to the development of ILD [45]. Thus, we detected the expression of IL-1, IL-6, and TGF-β in A549 cells treated with different concentrations of UA, and the results indicated that IL-1, IL-6, and TGF-β expression was elevated by UA in a dose-dependent manner, suggesting the considerable value of UA in ILD pathological processes.

However, the present study also had some limitations. First, higher UA levels could be correlated with heart failure, and some information on cardiac functions of enrolled patients with RA should be added and correlated to ILD pattern and UA levels. In addition, probably because of the limited number of included patients who had received ongoing treatment, we have not found statistically significant differences in drug therapy among different subtypes of patients with RA-ILD in the current findings. Therefore, we should include larger, more selected populations to further confirm whether there is some association of ongoing treatment with different RA-ILD subtypes in the future studies. Despite these limitations, our findings may have important clinical value. This study established that UA is strongly associated with RA, especially RA-ILD, which can be an important indicator for the onset and severity of RA-ILD. UA may also hold promise as a useful biomarker for RA-ILD.

## Data Availability

The datasets used and/or analyzed during the current study are available from the corresponding author on reasonable request.
